# Quantum Locality in Game Strategy

**DOI:** 10.1038/srep44730

**Published:** 2017-03-22

**Authors:** Carlos A. Melo-Luna, Cristian E. Susa, Andrés F. Ducuara, Astrid Barreiro, John H. Reina

**Affiliations:** 1 Centre for Bioinformatics and Photonics—CIBioFi, Calle 13 No. 100-00, Edificio 320 No. 1069, 760032 Cali, Colombia; 2 Departamento de Física, Universidad del Valle, 760032, Cali, Colombia; 3 Experimental Physics IV, University of Bayreuth, 95440 Bayreuth, Germany; 4 Clarendon Laboratory, Department of Physics, University of Oxford, Oxford OX1 3PU, UK

## Abstract

Game theory is a well established branch of mathematics whose formalism has a vast range of applications from the social sciences, biology, to economics. Motivated by quantum information science, there has been a leap in the formulation of novel game strategies that lead to new (quantum Nash) equilibrium points whereby players in some classical games are always outperformed if sharing and processing joint information ruled by the laws of quantum physics is allowed. We show that, for a bipartite non zero-sum game, input *local* quantum correlations, and *separable* states in particular, suffice to achieve an advantage over any strategy that uses classical resources, thus dispensing with quantum nonlocality, entanglement, or even discord between the players’ input states. This highlights the remarkable key role played by pure quantum coherence at powering some protocols. Finally, we propose an experiment that uses separable states and basic photon interferometry to demonstrate the locally-correlated quantum advantage.

In 1944, von Neumann developed a formal framework of game theory^1^, namely of understanding the dynamics of competition and cooperation between two or more competing parties that hold particular interests. In another seminal work, twenty years later, Bell discovered the intrinsic, fundamental nonlocal character of quantum theory^2^, the fact that there exist quantumly correlated (entangled) particles whose measurement gives results that are impossible in classical physics—the so-called violation of Bell inequalities^3^,^4^. Such Bell nonlocality and entanglement turned out to be of key relevance in the development of quantum information science and technology^5^. In fact, quantisation protocols for strategy games exemplify a physical process whereby entanglement or nonlocality are used as a fundamental resource^6^,^7^,^8^,^9^,^10^,^11^,^12^,^13^,^14^,^15^,^16^,^17^,^18^,^19^. This establishes a connection between game theory and quantum information and, as such, introduces the existence of certain advantages over the foregoing classical results^6^,^7^,^8^,^9^,^10^,^11^,^12^,^13^,^14^,^15^,^16^,^17^,^18^,^19^, and extends the set of cases that find solution to the interaction formalism^1^,^20^,^21^ into the quantum realm^6^,^7^. Such quantum features are reflected, e.g., in the increase of efficiency and payoffs, emergence of new equilibria, and novel game strategies which are simply not possible in the classical domain^12^,^17^,^18^,^19^. These achievements signalled an interest about the nature of such a quantum advantage, and introduced questions related to the properties of physical systems and the mathematical structure that underlies the novel game strategies^8^,^9^,^10^,^11^,^12^,^13^. Advantages of different kind became evident when quantisation rules were applied to different sort of games, and most of these scenarios pointed out quantum entanglement as a precursor of such effects^6^,^7^,^14^,^15^,^16^,^17^,^18^,^19^.

Furthermore, Bell nonlocality has been recently shown to provide an advantage when deciding conflicting interest games^8^,^9^,^10^. In this regard, and mostly inspired by strategies of this sort, the activation of quantum nonlocality has been put forward^22^,^23^,^24^. In particular, *k*-copy nonlocality or superactivation^24^, and activation of nonlocality through tensoring and local filtering^25^, although seminal for protocols based on nonlocality (e.g., quantum cryptography), are ultimately limited by the presence of entanglement^22^. This said, here we explore other kind of correlations that highlight local states as a possible resource for introducing a quantum advantage (see Fig. 1, shaded region). In particular, we ask whether there is, beyond entanglement or nonlocality, another underlying fundamental quantum feature as quantum coherence that warrants the emergence of the above-mentioned advantages. This consideration is also motivated by a recent experimental demonstration of a *zero*-*sum* game that exhibits a quantum gain for players that do not share entanglement^13^.

The Prisoners’ Dilemma (PD) game is a celebrated bipartite non-zero sum game in classical game theory^20^,^21^ whereby two parties, say Alice (*A*) and Bob (*B*), have to decide between two strategies in an independent way: to defect (*D*) or cooperate (*C*). The retribution to the players decision is conditioned to the pair of choices, as shown in Table 1. The classical PD game reveals the existence of a set of strategies from which unilateral movement of the players diminishes their payoff—a Nash equilibrium (NE)— and a set of strategies whereby the players simultaneously do best—a Pareto optimal^20^. The dilemma arises due to the choice problem between the equilibrium and the optimal gain.

The PD sum game has been extended to the quantum domain by Eisert *et al*.^7^, who proposed the use of initial maximally entangled states and unitary operators to define a strategy of purely quantum character that removes the decision dilemma^7^. Thus, the interaction between players can be cast in a quantum circuit that generates, via the action of a two-qubit operator 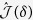, an initial state of the form:


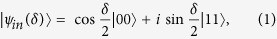


where 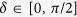. Here, the possible outcomes of the classical strategies *C* and *D* are assigned to the computational basis states |0〉 and |1〉, respectively, and the strategy space of each player has a Hilbert space structure that couples through a tensor product. In Fig. 2(a), the operator 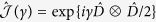 generates input entangled states^7^. After that, the players execute, unilaterally, their movements acting with the unitary parameterised operator (*i* = *A, B*)),


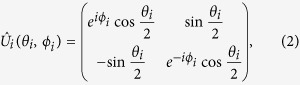


particularly, 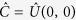 and 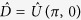 reduce to the classical strategies. Finally, an operator that destroys the entanglement generated by 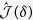 is applied before projecting the output state onto the usual 4-dimensional space basis, giving rise to a probability distribution above the four possible classical states, from which the expected payoff for each player is determined. The unitary operator Eq. (2) was introduced as a tool in the quantisation of non-zero sum games in ref. 7, the so-called two-parameter strategy set. However, it was pointed out that such an operator defined a set of strategies that did not consider the natural counterstrategy of the quantum strategy 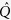 (see Results), and hence did not represent a general conclusion for other quantisation formalisms^26^. Such an observation was addressed in ref. 27, and incorporated to account for generalised two-parameter strategies in refs 28, 29, 30, also to consider the presence of dephasing^31^, and for multiplayer quantum games^14^,^32^. In spite of this restriction on the strategic space defined by the strategy 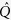
7, Eq. (2) has been used as the starting point in more elaborated constructions that allow for the analysis of game behaviour in the quantum domain^28^,^29^,^30^.

In this paper, we analyse the PD game and demonstrate that *local*, and even further, *separable* quantum input states suffice to achieve an advantage over any classical strategy. This result is in contrast with previous approaches to quantum games that consider entanglement or Bell nonlocality as required resources for achieving a quantum advantage^6^,^7^,^8^,^9^,^17^. Our finding is two-fold: First, we show that neither nonlocality nor entanglement can be regarded as the underlying fundamental properties responsible for the PD quantum advantage: we find purely discord-correlated states (zero entanglement) that also achieve such an advantage. Second, we show, by extending the set of Werner-like (*W*-*l*) states *ρ*_*W*-*l*_(*p*), that there exist (non-zero discord) input states for which the discord does not play any role at reaching this advantage. We also provide an optical setup that implements the locally-powered game strategy, and perform numerical experiments that demonstrate our findings. The analysis here presented is performed (although not limited) for the case of a two-parameter strategy set. In Discussion we address its extension to a more general three-parameter strategy set.

## Results

### Local quantum correlations as a resource in the PD game

**Figure 2:**
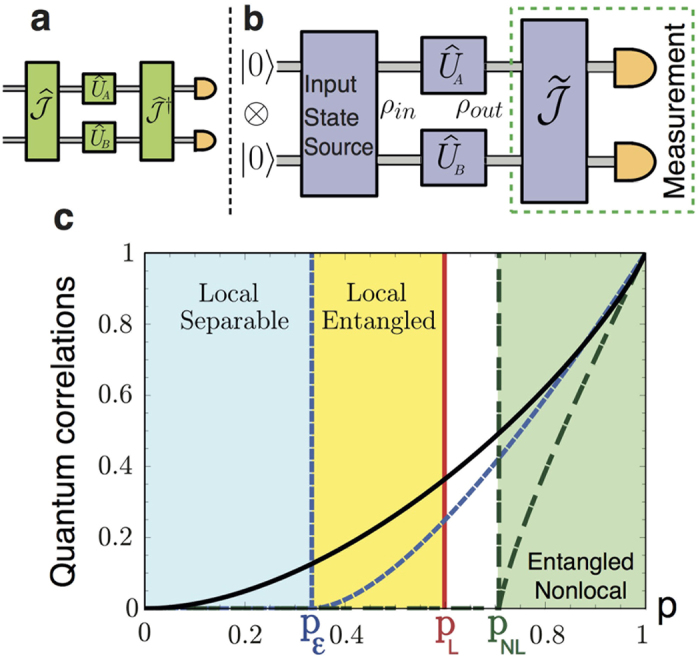
Quantum Prisoners’ Dilemma setup and classification of input correlations. (**a**) Eisert *et al*. two players game protocol^7^, (**b**) our setup uses a source of input 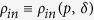 (e.g., Werner-like) states, one qubit gates to represent the players’ moves, and the measuring process (dashed rectangle). The measurement is taken as the projection onto the basis generated by 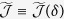 in the usual 4-dimensional basis, (**c**) quantum correlations of input *ρ*_*W*-*l*_(*p*) states: discord 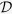 (solid-black), entanglement of formation 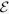 (dashed-blue), and CHSH-nonlocality (doubly-dashed green). justification = justified.

In contrast to the use of entangled states as a strategy for ‘quantising’ the PD game (Fig. 2(a))^7^,^17^, we explore a different feature and use the following input states (Fig. 2(b)) as the feeding resource for performing the quantum PD game:


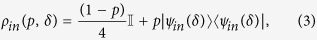


where 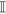 is the 4 × 4 identity matrix, and 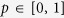 acts as a control of the statistical mixture *ρ*_*in*_(*p, δ*), and allows a direct comparison with the protocol of Fig. 2(a)^ ^7. In Fig. 2(b), the measurement process is made in a basis controlled by the same *δ* parameter, which allows the control of the degree of correlations that is ‘destroyed’ in the final step of the protocol, just before the projection onto the usual basis; i.e., the quantum operator 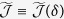 inside the dashed rectangle of Fig. 2(b) is defined such that 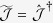 in the same way as the entangling operator of Fig. 2(a)^ ^7.

Every separable (non-entangled) state is local. However, there exist entangled states which are also local. For general two-qubit states of the form 
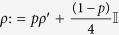
, 0 ≤ *p* ≤ 1, being *ρ*′ an arbitrary two-qubit state, a locality bound has been reported^33^. In our protocol, we identify 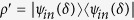 such that 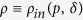, and hence the locality bound reads 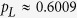; i.e., entangled states with *p* ≤ *p*_*L*_ are local (see the full local-entangled (LE) region, yellow area in Fig. 2(c)). Furthermore, we also account for the set of local, but separable (LS) states (see the blue Region in Fig. 2(c)).

In what follows, we first specialise to *W*-*l* states 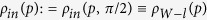 as inputs, and the correlation parameter *δ* is fixed to *π*/2 for the initial state, and only varied at the measurement. We then generalise our results to input states *ρ*_*in*_(*p, δ*), and consider the *δ* parameter being varied at both the input state and the final measurement process. For comparison, we also compute metrics to quantify quantum correlations such as discord 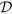, entanglement of formation 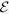, and CHSH-nonlocality; see Methods section for definitions.

### Quantum local PD payoffs for the Werner-like states

The quantum properties of the states *ρ*_*in*_(*p*) are shown in Fig. 2(c), where several distinctive regions can be identified: *local*-*separable* (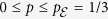), local-entangled (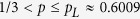), and entangled-nonlocal (
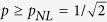
) states (see Methods section). Furthermore, the *W*-*l* states also highlight quantum correlations at zero entanglement (*p* ≤ 1/3)^34^, which are captured here by means of the discord^35^,^36^,^37^. Building on this, we take an approach that is not based on entangled^7^ or nonlocal^8^,^9^ input states. Instead, we choose local-separable 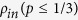 input states (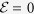, Fig. 2(c)), while the players’ quantum moves remain ruled by 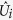, to test whether a quantum strategy based on such states removes the choice problem in the PD game. We calculate the corresponding PD payoffs for the *W*-*l* input states; for player *A* this reads:


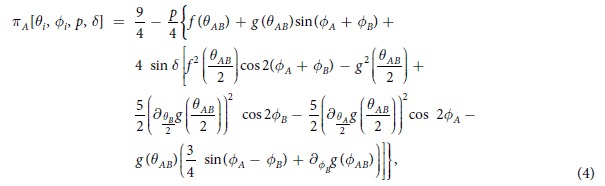


where 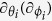 stands for the partial derivative with respect to 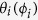, 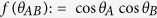 and 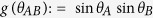. Player *B*’s payoff is obtained from equation (4) by exchanging indexes *A* and *B (i* = *A, B*).

**Figure 3:**
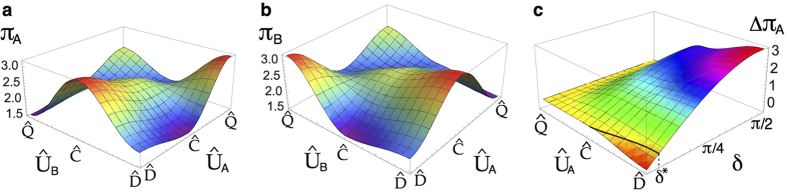
Players payoffs and Nash inequality for the quantum PD game. (**a**) Alice and (**b**) Bob’s payoff functions for the initial mixed-separable-discorded state *ρ*_*in*_(*p* = 1/3) as function of the strategy space; 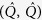, with 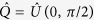, is the quantum strategy that removes the dilemma. (**c**) The left-hand-side value in equation (5), Δ*π*_*A*_, is plotted as a function of the players strategies and the measurement parameter *δ*. The Nash inequality takes positive values almost anywhere the surface, except at the red region below the black curve; e.g., for the particular strategy 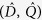, the inequality is not satisfied for *δ* < δ* = arcsin(1/7). Since *p* is just a global factor in equation (5), this behaviour holds even for input states with zero entanglement.

In Fig. 3, we plot the players’ payoffs as function of their strategies. We obtain a payoff distribution for which the solution criteria can be evaluated in order to find equilibrium strategies^20^; the classical solution criteria remain valid in the quantum context, and thus we introduce a 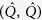 strategy, with 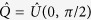, that removes the choice problem in the PD game^7^. This result arises ‘naturally’ by fixing *δ* = *π*/2 and *p* = 1 at both the input state and the measurement stage of our protocol. A thorough examination of the payoff functions, equation (4), reveals that whilst *p* controls the magnitude of the players’ payoff, *δ* modifies the shape of their distributions. This demonstrates that our *local* input state (*p* ≤ 1/3) keeps unaffected the equilibrium properties of the quantum version of the PD game as shown in Fig. 3(a,b) for the particular case *p* = 1/3 and *δ* = *π*/2. We then ask what happens to the Nash Equilibrium if both *p* and *δ* are modified at a given time, for which we next compute the corresponding Nash inequality.

### Nash equilibria of the game

In a finite game of normal form 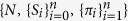, a strategy chain s* is NE to the *i*-th player if and only if 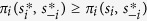, 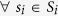, where *S*_*i*_ is the strategy space of player *i*, and *π* denotes the payoff function. We evaluate this criterion with respect to the quantum strategy 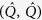, and for player *A* we obtain


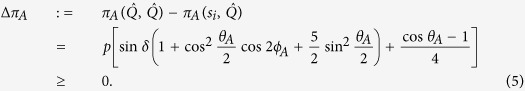


Note that 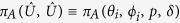. We reach the same result for Δ*π*_*B*_. This inequality does not depend on the value of *p*, and hence it holds even for zero entanglement input states; since we are interested in the quantum case, *p* = 0 is discarded. Thus, *δ* becomes a crucial factor when deciding whether this Nash inequality is satisfied. We highlight the novelty of the result equation (5): the quantum advantage, here reported, *does not* require neither the maximal entanglement condition *δ* = *π*/2 (nor any 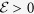 at all), nor that of nonlocality to be fulfilled; instead, the quantum strategy 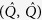 is a NE when the player *A* moves its strategy from 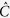 to 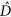, for 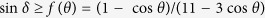. For the specific strategy 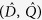, the critical value δ* is given by sin δ* = *f(π*) = 1/7, as explicitly shown in Fig. 3(c).

**Figure 4:**
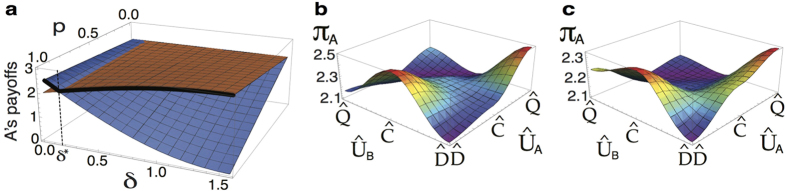
Nash equilibrium analysis for the Werner-like initial state: (**a**) Player *A*’s payoffs for 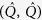 (brown-upper), and 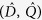 (blue-lower) strategies as functions of both the entanglement *δ* and the mixing *p* parameters. The black-solid curve at *p* = 1 shows the behaviour of the Nash equilibrium before and after the critical point δ* = sin^−1^ (1/7) (vertical-dashed line). Strategies space profile for player *A* payoffs with (**b**) *δ* = 0.2 > δ*, and (**c**) *δ* = 0.05 < δ* for the mixed input state *ρ*_*in*_(*p* = 1/3).

For clarity, in Fig. 4(a) we plot the player *A*’s payoff for the particular 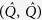 (brown-upper) and 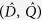 (blue-lower) strategies, in terms of the entanglement *δ* and mixing *p* parameters. The vertical-dashed line on the *p* = 1 plane marks the critical δ* at which the dominant strategy, i.e., the strategy giving a NE, changes. Hence, two regions arise for any *p* > 0: i) *δ* ≥ δ* = sin^−1^ (1/7), the quantum strategy 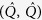 is the NE and Pareto optimal such that the choice dilemma is removed as can be seen for *π*_*A*_ in Fig. 4(b); ii) *δ* < sin^−1^ (1/7), the game does not present a strict NE but two at 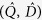 and 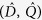, the payoff for player *A* is greater when choosing the former rather than the latter strategy, as shown in Fig. 4(c) (the opposite arises for player *B*–not shown). This asymmetry implies again a choice problem in the game such that the dilemma is not removed in this region. Figure 4(b,c) have been obtained for *p* = 1/3, and show that the advantage over any classical strategy is still achieved for separable states.

We stress that the quantum advantage in the PD game, here reported, is not a consequence of entanglement at the input state of the game. In general, as long as *ρ*_*in*_(*p*) can be generated, the quantum solution for removing the prisoners’ dilemma is achieved. This means that, for these particular input states, the quantum advantage in the non-zero sum game has been extended to a more general kind of quantum correlations, beyond entanglement, here quantified by the quantum discord. This is indeed emphasized, as mentioned above, by the quantum properties displayed by the states equation (3), as plotted in Fig. 2(c) for *δ* = *π*/2. Indeed, for *p* ≤ *p*_*L*_, *ρ*_*in*_(*p*) is local; furthermore, if the resource states *p* ≤ 1/3, then the input states are local-separable and not related whatsoever to either entanglement or nonlocality. In Fig. 2(c), we also find that discord is present in the whole *p*-region 0 < *p* ≤ 1. This said, a new question arises: how essential is quantum discord as a resource for the quantum advantage here reported? To address this question, we extend our analysis to input states with a more general structure, as given by *ρ*_*in*_(*p, δ*).

### Generalisation to input states *ρ*_*in*_(*p, δ*)

**Figure 5:**
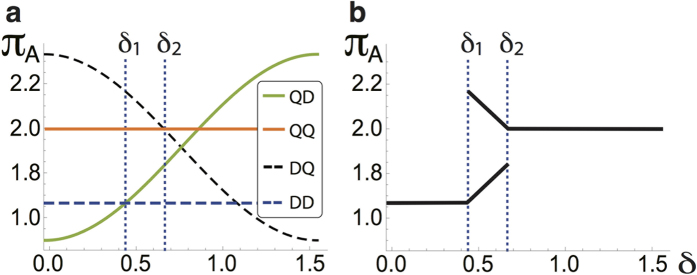
Payoffs for general states *ρ*_*in*_(*p, δ*): (**a**) the control of the initial state correlations, and 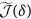 imply thresholds at 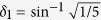, and 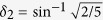, (**b**) strategies reaching the Nash equilibrium in the regions defined by *δ*_1_ and *δ*_2_.

If we now control the input state degree of correlations by varying *δ* in equation (3), Nash inequality holds as follows: for the strategy 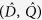 (or equivalently, for 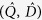), 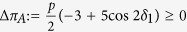, and for the strategy 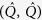, 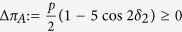. Three regions arise, as indicated in Fig. 5(a,b), by means of 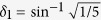, and 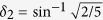. The payoff for the players in the 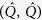 strategy will be constant in the same way that for the 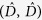 strategy. This behaviour is crucial for values greater than *δ*_2_ because the Nash equilibrium is reached, and the dilemma is removed. The key parameters *δ*_1_ and *δ*_2_ obtained here for the considered mixed states coincide with those reported by Du *et al*.^17^ for just pure states. This is because *p* only affects the size but not the shape of the payoff functions. For example, by computing the Nash inequality for *A*’s payoff in the 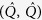 and 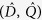 strategies, *p* holds as a global parameter and does not affect the bounds of the inequality. Finally, we show that by considering the *W*-*l* states *ρ*_*in*_(*p*) and just controlling the degree of correlations in the final operator 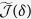, we reach the quantum advantage which removes the game dilemma for *δ* values smaller than those reported before^17^, and, crucially, δ* < *δ*_2_, even for separable states.

**Figure 6:**
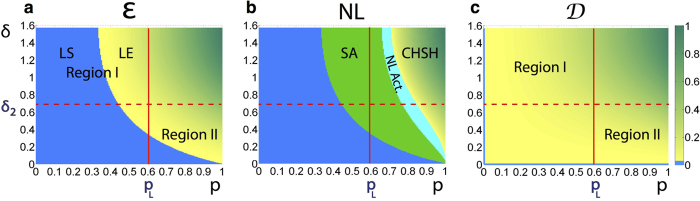
Quantum properties of the input states *ρ*_*in*_(*p, δ*) and quantum advantage bound. As a function of *δ* and *p*, we plot: (**a**) Entanglement of Formation (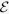): the blue area represents the set of separable and therefore local states, and all the states 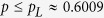, as depicted by the vertical line *p* = *p*_*L*_, are also local (for the joint correlation)^33^; these allow the identification of the local-entangled (LE) region of states, (**b**) non-locality (NL) properties: CHSH inequality violation, *k*-copy nonlocality or superactivation (SA) of non-locality (green-solid area), and activation of non-locality (NL Act.) through tensoring and local filtering (cyan-solid area), and (**c**) quantum discord (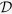): the Region I (*δ* ≥ *δ*_2_, *p* ≤ *p*_*L*_, upper left rectangles) spans non-zero discord states that even though local, allow a quantum advantage; the Region II (*δ* < *δ*_2_, *p* > *p*_*L*_, lower right rectangles) portrays non-local and local non-zero discord states for which the choice dilemma is not removed. The bound 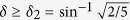, for which the quantum advantage holds, is depicted by a horizontal red-dashed line.

For the sake of completeness, we analyse the quantum advantage in the PD game, i.e., the two regions defined by the *δ*_2_ bound, from which the quantum 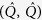 strategy removes the dilemma, in terms of the quantum properties of the input *ρ*_*in*_(*p, δ*) states. We plot the entanglement of formation (Fig. 6(a)), non-locality given by CHSH inequality violation, *k*-copy nonlocality (SA)^24^, and activation through tensoring and local filtering^25^ (NL Act.) (Fig. 6(b)), and quantum discord (Fig. 6(c)), all of them as functions of the correlation *δ*, and mixing *p* parameters (see the Methods section for definitions). We distinguish two principal regions in Fig. 6: Region I (*δ* ≥ *δ*_2_, and *p* ≤ *p*_*L*_, upper left rectangles) in which it is possible to find local-entangled states, and more interestingly, separable states which are able to remove the choice dilemma as they admit the quantum 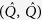 strategy to be the NE and Pareto optimal (see Fig. 5). This implies that there exist local quantum states that can be seen as a powering resource for performing quantum strategies that outperform any possible classical strategy in a PD game. In Region II (*δ* < *δ*_2_, and *p* > *p*_*L*_, lower right rectangles), there are states with different nonlocal properties (Fig. 6(a,b)) admitting no quantum advantage for removing the choice dilemma in the PD game. It is worth pointing out that the nonlocal properties here analysed are bounded by entanglement, i.e., all of them cover sets of states smaller than or equal to the one representing the entangled states. On the other hand, Fig. 6(c) clearly shows that even for some discord-correlated states, the dilemma is not removed in this region, hence explicitly showing the existence of non-zero discord states that exhibit no quantum advantage. Thus, discord on its own cannot be regarded as a fundamental measure (beyond entanglement) that underpins the quantum advantage.

### Experimental proposal for demonstrating the locally-correlated quantum advantage

**Figure 7:**
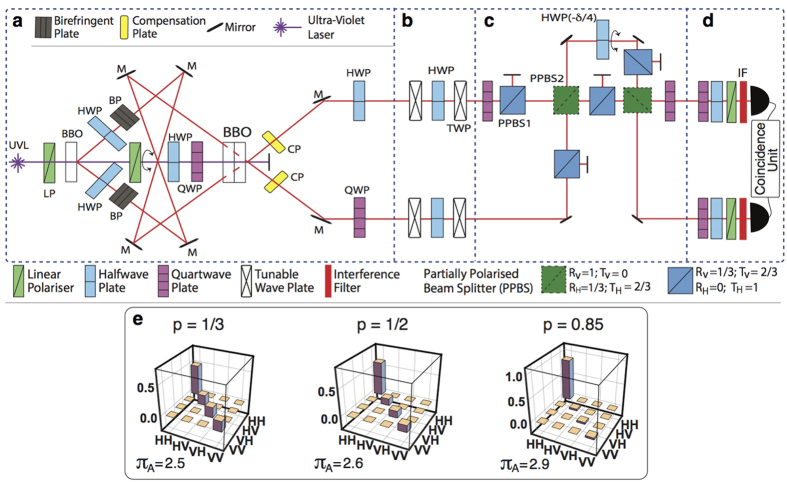
Experimental setup to demonstrate the local quantum advantage in the PD game. Dashed boxes: (**a**) protocol that generates the input states starting from |*VV*〉: a Werner state is created and successive applications of an *X* and a *π*-phase gates lead to *ρ*_*in*_(*p*) (equation (3)), (**b**) the individual action of the players on each qubit, 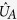 and 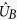, (**c**) implementation of the quantum operations *π*/2-phase shift, C-NOT, 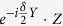, C-NOT, *π*/2-phase shift (*Y* and *Z* are the usual Pauli gates), (**d**) the standard tomography protocol to reconstruct the final state which gives the players payoffs, (**e**) expected tomographies and player *A*’s payoffs for separable (*p* = 1/3), local-entangled (*p* = 1/2 ≤ *p*_*L*_), and non-local (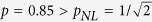) input states; *δ* = *π*/2, and chosen strategy 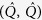.

The described quantum PD game based on local input states can be experimentally tested, e.g., by optical means. In Fig. 7 we give a setup that uses an optical encoding of horizontal (|*H*〉) and vertical (|*V*〉) polarisation states as qubits. The experimental process is divided into four main steps: preparation of the initial state (Fig. 7(a)), setting the players’ strategies (Fig. 7(b)), tailoring a quantum operation on the output state (Fig. 7(c)), and detection of the game’s result via quantum state tomography (Fig. 7(d)). The detailed implementation of these four steps is described in the Methods section.

In Fig. 7(e), we have performed a numerical experiment in order to obtain the Alice’s payoffs based on the local-separable *ρ*_*in*_(*p* = 1/3), local-entangled *ρ*_*in*_(*p* = 1/2), and the non-local *ρ*_*in*_(*p* = 0.85) states, for the 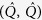 strategy. In so doing, we have considered the following feasible experimental parameters: laser wavelength *λ* = 351 nm, converted central wavelength *λ*_0_ = 702 nm, retardation length 153*λ*_0_ and 306*λ*_0_, spectral bandwidth Δ*λ* = 10 nm, and birefringent plates with a constant difference of *π*/2 between them for their rotation angles. These simulations are in excellent agreement (not shown) with the result that is obtained by simply following the abstract circuit of Fig. 2(b). We stress that our results show that the PD quantum advantage is achieved in the three different considered scenarios regardless the nonlocal or entanglement features of the considered quantum input states.

## Discussion

Purely *local* and/or *separable* input quantum states have been harnessed as a resource in the PD game, and we have shown that such a strategy gives a clear advantage over the original bipartite non-zero sum game that makes use of just classical resources. In particular, we have also shown that neither entanglement nor any nonlocal property is strictly required at the input of the game in order to achieve a quantum 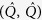 strategy that removes the PD dilemma and hence outperforms any classical strategy. First, our results have been explored for Werner-like states with known nonlocal properties, but also extended to a more general class of correlation-parameter-dependent states (equation (3)). Second, we have shown that within the set of discord-correlated states, there exist some states for which the PD choice problem is not removed, thus implying that quantum discord is neither a necessary condition for achieving the above-described quantum advantage. These results point out the interesting and relevant role played by separable quantum states (and therefore locality) when designing quantum strategies that outperform those based on classical resources, and suggest that such a key resource actually arises from basic quantum interference mechanisms, i.e., quantum coherence, whose description as a physical resource is a rapidly growing conceptual development^38^.

Even though we have focused in the PD version in which both players choose their strategies from the particular set of two-parameter strategies (Eq. (2)), we next demonstrate that our findings can be extended to more general scenarios^28^,^29^,^31^. In the case of a three-parameter set of strategies, it is known that there is no pure strategy being a Nash equilibrium because for every strategy of Alice, there exists a counterstrategy available for Bob^26^. However, it is possible to have a mixed strategy Nash equilibrium that is non unique^28^. If we assume that Alice and Bob can carry out the following set of strategies^28^,^31^


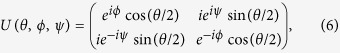


they can reach a mixed equilibrium when Alice chooses *U*(0, 0, *ψ*) and *U*(0, *π*/2, *ψ*) with the same probability, and Bob chooses *U(π, ϕ*, 0) and *U(π, ϕ, π*/2) with the same probability^28^,^31^. Despite the lack of uniqueness of the above equilibrium, a unique equilibrium can be found by applying the local point effect^39^. However, our main aim is to show that our findings can be extended to this scenario and that the major analysis on the role of quantum (e.g. Bell non-local, entangled local, or just local) correlations holds. For doing so, we demonstrate that the optimality of the mixed quantum strategies with respect to the classical strategies behave in the same way as for the case of the two-parameter strategy considered above.

Assuming that Alice and Bob choose the aforementioned quantum mixed strategies and apply them to our initial input state *ρ*_*in*_(*p, δ*), Alice’s payoff becomes:


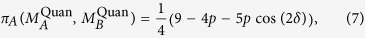


where 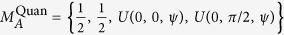 and 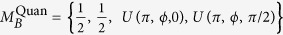. Now, assuming that Alice decides to play the classical mixed strategy 
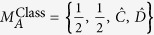
 instead of the above quantum mixed strategy, while Bob keeps his quantum movement, it is straightforward to show that the inequality 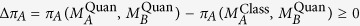 leads to


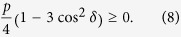


This comparison between the quantum mixed strategies and the classical one shows that the new payoff inequality does not depend on the input state parameter *p*. Hence, our findings regarding the actual role played by quantum (local or non-local) correlations in game behaviour remain valid for this more general scenario. Finally, we remark that a description of the focal equilibrium available in the considered three-parameter strategy is beyond the scope of this work and is left as a further development for the interested reader.

The simulated experiment for computing the tomography of the final states of the game, as well as their associated payoff functions (Fig. 7), show that our findings are amenable (although not restricted) to being tested with current photonics technology, as the involved optical devices follow well established, achievable laboratory parameters. We stress that since our PD protocol makes use of disentangled states as captured by equation (3), their optical generation, via the component 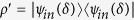 of the mixed state *ρ*_*in*_(*p, δ*), can be facilitated by the fact that ‘imperfect’ *W*-*l* states are more likely to be obtained in the laboratory, in addition to the fact that different (*p, δ*)-states can be achieved by varying the tilt angle of the second BBO, and by modifying the length of the compensator plates in Fig. 7, thus facilitating the photon interferometry here devised to demonstrate the quantum advantage.

We remark that we have mainly focused on generating the sufficient conditions for the purely quantum strategy 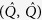 to solve the dilemma in a realistic scenario. This is why we consider an initial state perturbed by a white noise, as well as a non maximally entangled measurement basis. Furthermore, we extend our discussion to the more general case in which not only the entanglement of the measurement basis is varied, but also the entanglement in the *ρ*′ component of the input state, i.e., we consider the variation of the same correlation parameter *δ* at both the beginning and the end of the PD game. Related results for the threshold in the NE inequality have been reported^40^, but for some restricted input entangled states.

## Methods

### Quantum nonlocality-related properties of the game input states *ρ*_*in*_(*δ, p*)

A general finite-dimensional bipartite *AB* system is represented by a density matrix or quantum state 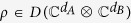, with *d*_*A*_, *d*_*B*_ ≥ 2, where 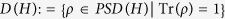 stands for the set of density matrices of the complex Hilbert space *H*, with *PSD* the set of *positive semidefinite* complex matrices, i.e., the matrices *ρ* such that 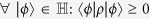. Here, we focus on the quantum properties of our two-qubit input states *ρ*_*in*_(*δ, p*) as shown in Fig. 6, where we have emphasised the locality region (*p* ≤ *p*_*L*_) which is limited by the value 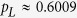 (vertical line), according to the best known bound^33^. This locality means that a Hidden Variable Model can be found to reproduce the same joint correlation of Alice and Bob 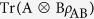 predicted by quantum mechanics, where *A* and *B* are observables on the state of Alice and Bob, respectively^33^. The aforementioned nonlocal quantum features of the input states plotted in Fig. 6 for performing the PD game are described as follows.

### Entanglement

We use the entanglement of formation 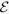 as a bipartite entanglement metric^41^. Let *ρ*_*AB*_ be the quantum state shared by Alice and Bob; the entanglement of formation of *ρ*_*AB*_ reads^41^:


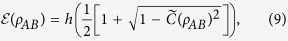


where 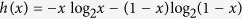 is the binary entropy, and 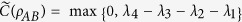 the *concurrence*. The *λ*_*i*_’s refer to the square root of the eigenvalues belonging to the auxiliary operator 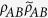 arranged in decreasing order, and 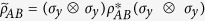
41.

### Discord

The role played by all the quantum correlations in the PD game is cast by means of the quantum discord 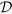, a metric defined as the minimum difference between the quantum version of two classically-equivalent ways of defining the mutual information^35^:


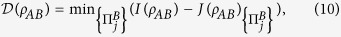


where 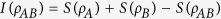 is the quantum mutual information, 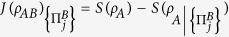 is the conditional mutual information associated to the state of the subsystem (say *A*) after the state of the subsystem (say *B*) has been measured (applying POVM operators 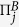), 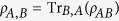, the conditional entropy 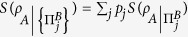, with probability 
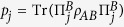
, and the density matrix after the measurement on *B* is given by 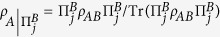
35,36,37.

### CHSH-Nonlocality

Given 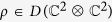, the Clauser-Horne-Shimony-Holt (CHSH) inequality^3^ considers two dichotomic observables per party (eigenvalues ±1), namely (*A*_1_, *A*_2_, *B*_1_, *B*_2_), and it takes the form:


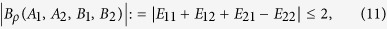


where 
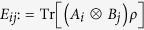
, *i, j* = 1, 2. It is said that *ρ* violates the CHSH inequality if and only if 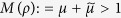, where *μ*, 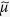 are the biggest two eigenvalues of the matrix 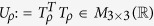, with 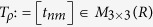, with elements 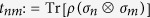, *σ*_*k*_, *k* = 1, 2, 3, the Pauli matrices. This arises from the fact that 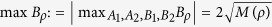
42. Then, using the Tsirelson’s bound^43^, 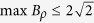, it follows 0 ≤ *M(ρ*) ≤ 2, showing nonlocality in the interval 1 < *M(ρ*) ≤ 2. Instead of *M(ρ*), we could work with 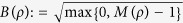 given that, for pure states, the former equals the concurrence: 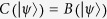
44. However, in order to have a direct comparison with 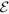, in Fig. 6(b), we compute nonlocality through the CHSH inequality, by plotting 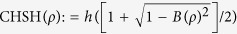, where *h(x*) is the binary entropy.

### *k*-copy nonlocality (superactivation)

Given 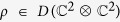, if *ρ* is useful to teleportation then is *k*-copy nonlocal^45^, i.e., *ρ* admits *superactivation* of nonlocality^24^. Usefulness to teleportation can be numerically tested by computing the Fidelity of Teleportation, which can be written as 
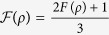
, where *F* denotes the Fully Entangled Fraction^46^, which for two qubits reads 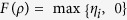, with *η*_*i*_’s the eigenvalues of the matrix *M* = [*M*_*mn*_], of elements 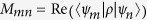, and {|*ψ*_*n*_〉} the so-called magic basis 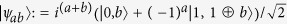
47. *ρ* is useful to teleportation if and only if 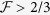
46. In our case, as shown in Fig. 6(b), the set of states that can be super-activated coincides with the whole set of entangled states (although this fact does not hold in general).

### Activation of nonlocality through tensoring and local filtering

Given 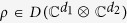 for subsystems *A* and *B* with arbitrary dimensions *d*_1_ and *d*_2_ respectively and, defining *P*_*CHSH*_ as the set of states that do not violate the CHSH inequality, even after local filtering, we say that 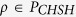 admits *activation* of nonlocality through tensoring and local filtering^25^ if there exists a state 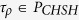 such that 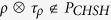. The latter is equivalent to have 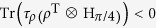, with 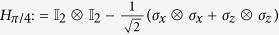, with T denoting transposition^25^. A theorem^25^ establishes the existence of such matrices *τ*_*ρ*_ in the space 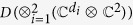 for any entangled *ρ*. Although the existence of such a matrix *τ*_*ρ*_ is already guaranteed, the theorem does not explicitly tell us how to calculate it. We have numerically tested this activation^25^ by looking for a state *τ*_*ρ*_ with positive partial transpose with respect to the first subsystem, 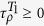 (say 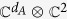)^48^,^49^, since this implies 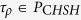
50. Thus, we solved the optimisation problem 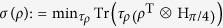 under constrains 
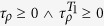
25. Even though the considered activation of the nonlocality region covers the whole entangled states^25^, the region for which we are indeed able to find the ancillary matrix required for the activation is represented by the cyan solid area (which covers the CHSH inequality violation region) in Fig. 6(b).

### All-optical setup to demonstrate the locally-powered quantum advantage

In Fig. 7(a), a laser beam is sent, through a linear polariser defining the input, to the first nonlinear crystal (BBO-*β* barium borate type I) as |*H*〉. After the first BBO crystal the state holds |*VV*〉, we then use a couple of half-wave plates (HWP) rotated azimuthally *θ* = *π*/8 to apply a Hadamard gate to each qubit such that |*V*〉 is transformed into 
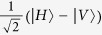
, and hence a superposition of all basis states is generated^51^. Sequentially, a birefringent environment (a set of quartz or BBO plates) is applied to each photon path and tuned to the maximum decoherence, which only affects the off-diagonal elements of the density matrix^51^, thus setting the state *ρ*_1_ = *I*/4. After the first BBO crystal, the non-converted remaining light is transformed into 
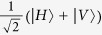
 by a HWP and pre-compensated through a quarter-wave plate (QWP), then directing it to a second set of BBOs which comprises a couple of crystals with mutually-perpendicular optical axes to create a maximally entangled state^52^. By combining the rays that passed through the first and second BBOs, the Werner state 
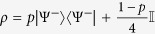
 is produced, where 
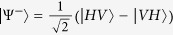
 is a Bell basis state. We next apply a *σ*_*x*_-gate to the upper path through a HWP with *θ* = *π*/4 as a rotating angle, thus transforming 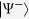 into the 
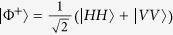
 Bell state. Then, a *π*/2-phase shift gate is applied to the lower path using a QWP with *θ* = 0 and hence producing *ρ*_*in*_(*p, π*/2) (equation (3)), the input state of our quantum PD game. Here, *p* can be tailored by allowing control of the intensity ratio between the converted light in the first BBO and the converted light in the second BBOs^51^: 0 ≤ *p* ≤ 1 could be tuned by adjusting the rotation angle of a linear polariser with respect to its optical axis located on the unconverted path just after the first BBO; *p* can then be measured from the total irradiance (*I*_*T*_) after the second conversion, and the partial irradiance (*I*_*P*_) of the light converted in the first BBO, as 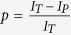. Thus, the local *ρ(p* = 1/3) input state can be achieved by setting *I*_*P*_ = 2*I*_*T*_/3.

Figure 7(b) implements the actions of the players (operator 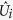 in equation (2)) by means of a set of wave plates, where the phase *ϕ* corresponds to the retarding angle of each plate, i.e., *ϕ* = *π* for a HWP, and *ϕ* = *π*/2 for a QWP. The angle *θ* corresponds to the perpendicular rotation of the centre half wave plates, referred to their optical axis. In Fig. 7, we use a special kind of wave plate that does not have a defined angle *ϕ*, the so-called tunable wave plate (TWP), which allows us to generate 0 ≤ *ϕ* ≤ 2*π*. In Fig. 7(c) we start from the output state *ρ*_*out*_ (Fig. 2(b)) generated in the previous step. To test the NE inequality, equation (5), we apply a phase gate by means of a QWP on the lowermost path, and resort to the use of a quantum Controlled-NOT gate which comprises a set of three partially polarised beam splitters (PPBS)^53^,^54^,^55^,^56^, where the two PPBS1s completely transmit the photons with |*H*〉 and 1/3 of the |*V*〉 polarisation, while the PPBS2 completely reflects |*V*〉 and 1/3 of the |*H*〉 polarisation. Then, a HWP with 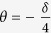 acts as a controller of the *δ* parameter over the control output of the first C-NOT gate (uppermost path), and additionally, a symmetrical arrangement of another C-NOT, and a QWP(*θ* = 0) completes the quantum operator 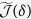. Finally, the measurement process is depicted in Fig. 7(d); a standard quantum state tomography protocol^57^, which requires a set of 16 measures is performed in order to obtain the final state of the system and the result of the game^58^.

## Additional Information

**How to cite this article:** Melo-Luna, C. A. *et al*. Quantum Locality in Game Strategy. *Sci. Rep.* **7**, 44730; doi: 10.1038/srep44730 (2017).

**Publisher's note:** Springer Nature remains neutral with regard to jurisdictional claims in published maps and institutional affiliations.

## Figures and Tables

**Figure 1:**
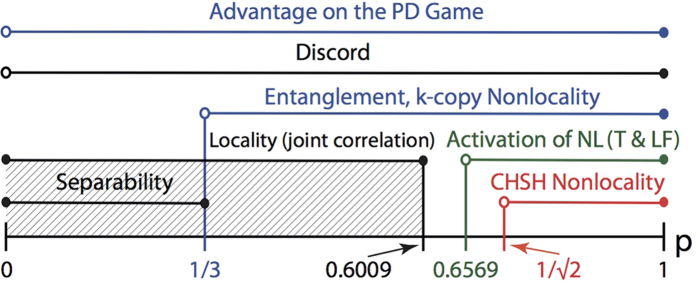
Some quantum properties for two-qubit Werner-like states. The schematics highlights locality (for the joint correlation), entanglement, CHSH-nonlocality, *k*-copy nonlocality, activation of nonlocality through tensoring and local filtering, and discord, for the Werner-like states 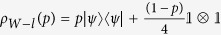, 
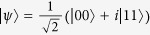
, 0 ≤ *p* ≤ 1. These states can lead to a PD game advantage in the whole *p*-region.

**Table 1:** Payoff matrix for the PD game.

Alice\Bob	*C*	*D*
*C*	(3, 3)	(0, 5)
*D*	(5, 0)	(1, 1)

The first (second) entry in the parenthesis denotes Alice’s (Bob’s) payoff. In the classical game, the strategy (*C*, *C*) defines a Pareto optimal (joint maximum gain), and (*D*, *D*) a Nash equilibrium.
